# Comparison of the effects of esketamine, sufentanil, or lidocaine combined with propofol on tussis reflection during upper gastrointestinal endoscopy: study protocol for a randomised, two centre, three-blind, controlled trial

**DOI:** 10.1186/s13063-023-07812-0

**Published:** 2024-01-04

**Authors:** Hai-jun Hou, Lu Liu, Ming Tian, Fu-shan Xue

**Affiliations:** 1 Department of Anesthesiology, Beijing Friendship Hospital, Capital Medical University, Beijing, 100050 China; 2 Department of Day Surgery Center, Beijing Tiantan Hospital, Capital Medical University, Beijing, 100070 China

**Keywords:** Upper GI, Tussis reflection, Propofol, Esketamine

## Abstract

**Background:**

Tussis, which increases the incidence of airway spasm, aspiration, nausea, and vomiting, is a common complication faced during upper gastrointestinal (GI) endoscopy. However, sedatives and analgesics exhibit inhibitory actions against airway reflexes to different degrees. Our assumption is a combination of propofol and small doses of sufentanil, esketamine, or lidocaine, especially the combination of propofol and esketamine, might reduce tussis incidence.

**Method:**

The study will be performed as a randomised controlled three-blind, two-centre trial. Patients undergoing upper GI endoscopy, ≥ 18 years old, with American Society of Anesthesiologists (ASA) classification I–III will be randomised to four groups: P group (single administration of propofol), P + S group (administration of propofol and sufentanil in combination), P + K group (administration of propofol and esketamine in combination), and P + L group (administration of propofol and lidocaine in combination) (*N* = 100 per group). The primary endpoints include the frequency of tussis, nausea and vomiting, and/or body movements observed at the insertion of the endoscope into the pharyngeal cavity or within 5 min of endoscope insertion. Secondary outcomes are recovery assessment, patients’ and endoscopists’ satisfaction with the procedure, MMSE scores, MET scores, sleep condition, and the number of sedation-related events. Data on sedation-related events are collected by recording of vital signs. Satisfaction parameters and mental states are collected by means of questionnaires and evaluation scales before and after the procedure and on different following days.

**Discussion:**

Esketamine can reduce tussis occurrence with good tolerability and relax the bronchus and also provides high clearance rates and low possibility of adverse reactions. We aim to demonstrate that the combination of esketamine with propofol for sedation in patients subjected to upper GI procedure is nevertheless superior to only administration of propofol or a combination of propofol with other anaesthetics, such as opioids or lidocaine.

**Trial registration:**

ClinicalTrials.gov. NCT05497492, Registered 09 August 2022.

**Supplementary Information:**

The online version contains supplementary material available at 10.1186/s13063-023-07812-0.

## Introduction

The endoscopic technique is the most widely utilised method for the diagnosis and treatment of upper gastrointestinal (GI) diseases and is vital in clinical cases for the screening and treatment of upper GI tumours. However, without anaesthesia, upper GI endoscopy is painful for patients due to possible side effects, such as nausea, retching, tussis, and dyspnoea, caused by frequent stimulations on the throat resulting from the endoscope during comprehensive and careful observation. This discomfort is highly intense for patients with chronic pharyngitis and a drinking and smoking history. A low tolerance of the patients to general upper GI endoscopy often leads to poor results and low willingness for repeated examination. With the development of recent anaesthetic techniques and increasing demand for comfortable medical services, the promotion and popularisation of endoscopy under anaesthesia has become an inexorable trend; thus, patient discomfort is alleviated to the maximum extent during examination [1].

Tussis, which increases the incidence of airway spasm, aspiration, nausea, and vomiting, is a common complication faced during upper GI endoscopy and a protective cough reflex. The airway epithelium is highly sensitive to mechanical and chemical stimuli. The entrance of the endoscope in the oral cavity induces mechanical stimuli to the respiratory tract, activates the cough receptor in the airway, and triggers tussis by activating cough through n-methyl-d-aspartate (NMDA) receptors [2]. However, sedatives and analgesics exhibit inhibitory actions against airway reflexes to different degrees. To date, propofol has been widely utilised for sedation during endoscopy due to its advantages, such as the rapid onset of action; short action time; widespread inhibitory functions on the central nervous system; strong effects on suppressing the contraction of GI smooth muscles, antagonising vomiting reflex; and reducing cough, body movement, and post-operative headache [3, 4]. By contrast, as a single drug, propofol may confer insufficient sedation, weak inhibition against tussis reflection, and short effective duration, thereby necessitating compensation through extra doses. However, repetitive increases in the dose result in the prolongation of postoperative revival, elevated risks of postoperative respiratory depression and hypoxemia, and prolonged hospital residence for postoperative recovery [5, 6]. A study reported reflex reactions induced by stimulating the throat under only propofol anaesthesia [7], which limited the probability of the single use of propofol in endoscopy. Researchers have reported sound sedative effects, inhibitory actions against throat reflection, and stress reactions achieved by the combined use of propofol and small doses of opioids (sufentanil: 0.05 μg/kg or 2–5 μg), which palliates intraoperative pain and discomfort, reduces the postoperative recovery time [8], and is extensively utilised for painless upper GI endoscopy. However, propofol may result in hypotension, bradycardia, and respiratory depression [9]. Research [10, 11] shows that compared with the combination of opiates/benzodiazepines or the use of propofol alone, propofol is more effective and safer when used in combination with ketamine or opiates (sufentanil) and other adjuvants for procedural sedation/anaesthesia.

Esketamine has irreplaceable advantages in maintaining autonomous respiration and sympathetic nervous system-like characteristics. Compared with the combination of opiates and propofol, the combination of ketamine and propofol is preferable, because the latter may increase the possibility of respiratory depression. Esketamine, the d-enantiomer of ketamine with pharmacological actions similar to ketamine’s actions and twice its action intensity provides high clearance rates and low possibility of adverse reactions, which are dose dependent [12]. Small doses of esketamine (recommended dose = 0. 15 mg/kgp [13]) can reduce tussis occurrence with good tolerability. In addition to its role in antagonising NMDAR (the n-methyl-d-aspartate receptor) widespread in the airway, throat, and lungs, esketamine directly acts on airway smooth muscles to expand bronchi through voltage-dependent L-type calcium channel and thus prevents and represses tussis reflection [14]. However, the sympathomimetic action of esketamine contributes to the transient increase in blood pressure and heart rate, whose superimposition with the endoscope insertion–stimulated sympathetic reflex exerts additional burden on the heart and is detrimental to patients with potential myocardial ischaemia [15]. However, when used in combination with propofol, esketamine can not only abolish the suppressive action of propofol on circulation and breathing but also lead to a decrease in propofol doses; thus, esketamine became increasingly popular in clinics [13]. Considering the previously reported evidence about these complementary effects of esketamine as an adjunct to propofol, the combined use of esketamine and propofol may be a promising approach that could reduce the risk of oversedation of propofol in gastrointestinal endoscopy.

Intravenous administration of lidocaine blocks the Na^+^ channel on the sympathetic cell membrane and inhibits the function of the sympathetic adrenal system, transduction of neural signals, and tracheal mechanical stimulation of the function of mucosal sympathetic receptors to prevent tussis reflection. Considerable studies have validated the efficacy of the intravenous injection of 1–2% lidocaine in anaesthesia during upper GI endoscopy; however, the myocardial depression and high individual risks of anaesthetic toxicity necessitate further explicitness of security in applications.

The best approach to analgesia and sedation during upper GI endoscopy is controversial; thus, the development of an appropriate sedative/analgesic strategy that can influence the examination quality, patient cooperation, and degree of satisfaction towards anaesthesia is imperative. From the preliminary observations of clinical trials, we assumed that compared with single-use of propofol, a combination of propofol and small doses of sufentanil, esketamine, or lidocaine, especially the combination of propofol and esketamine, might reduce tussis incidence. To verify our hypothesis, this study expounded the effects of different drugs combined with propofol on tussis reflection in upper GI endoscopy.

## Methods and analysis

### Study design

This paper presents a prospective, randomised, controlled, three-blind, two-centre study registered in the Chinese Clinical Trial Registration Center. The trial will be conducted at Beijing Friendship Hospital and Beijing Tian Tan Hospital, Capital Medical University, China. We have used the SPIRIT reporting guidelines and completed SPIRIT checklist (Additional file 2). Hence, this study enrolled 400 patients (200 patients per centre) from October 2022 to April 2023. Before the study starts, all the staff took a course so they can explain more clearly to the patients, which helped us to get enough participants. The random sequence generator of STATA MP 14 software generated random numbers and divided them into four groups. Patients will be randomly divided into four groups in the ratio of 1:1:1:1. For each selected patient, the researchers drew an envelope to determine their grouping, but the patients were not informed of the grouping. Except for propofol, clinical researchers injected transparent solution into the syringe in random order in the same bottle containing the code. The researchers who conducted randomisation and blinding procedures did not participate in subsequent studies. Other researchers were not informed about the grouping of research and experimental drugs. To ensure the concealment of the allocation, the randomisation results were sealed until the end of the study. The trial auditing process was conducted by an independent team. Whether a centre underwent a monitoring or auditing visit was determined based on scheduling and availability of the auditor, their proximity to each site, and site convenience. We sometimes scheduled the timing of auditing visits for new research staff or following site initiation visits for a different trial led by the Methods Center to reduce travel-related costs. Auditing was conducted in-person.

### Eligibility criteria

Patients who (1) undergo elective upper GI endoscopy under deep propofol sedation, (2) were ≥ 18 years old, (3) meet the classification I–III of American Society of Anesthesiologists (ASA), and (4) give written informed consent were selected.

### Exclusion criteria

Patients with the following medical history criteria will be excluded from the study: (1) allergic reaction to planned medication; (2) gravis myasthenia; (3) history of psychological problems or psychiatric disease; (4) morbid obesity/obstructive sleep apnoea; (5) acute upper respiratory infections; (6) asthma at acute stage; (7) history of unregulated or malignant hypertension, significant ischemic heart disease, severe arrhythmia; (8) uncontrolled hyperthyroidism; (9) severe cardiac, liver, and kidney dysfunction and coagulation disorders; (10) acute upper GI haemorrhage with shock; (11) severe anaemia; (12) GI obstruction with gastric retention; (13) seizure disorders, long-term history of sedative and analgesic drug use, and history of allergy; and (14) increased intracranial pressure and high intraocular pressure.

### Patient and public involvement

The public or patients will not be involved in the design, conduct, reporting, or dissemination plans of this research.

### Randomisation and blinding

Randomisation will be performed as follows. Based on different study centres (two groups), the patients will be classified into four groups: P group (single administration of propofol), P + S group (administration of propofol and sufentanil in combination), P + K group (administration of propofol and esketamine in combination), and P + L group (administration of propofol and lidocaine in combination) (*N* = 100 per group). The random sequence generator of the STATA MP 14 software generated random numbers and divided them into four groups. Only the nurse (A) who is responsible for preparing the trial drug knows the grouping information automatically generated by a computer. She (A) will generate the allocation sequence. The chief anaesthesiologist (B) will enrol patients and assign participants to interventions. Computer-generated random grouping numbers will be printed and placed in separate sealed envelopes. After meeting a participant who meets the inclusion criteria, the anaesthesiologist will assign the newly recruited participant to a group according to the number provided in the envelope. Both the anaesthesiologists and patients are unaware of the treatment plan. Anaesthetic drugs will be prepared and labelled with numbers by nurses and injected by the anaesthesiologists. The anaesthesiologists will record the physiological characteristics of each patient. In emergencies, the nurses can provide the anaesthesiologists the information of drugs to ensure clinical safety. The designed blinding method is as follows: This study will use the three-blind method to ensure the objective evaluation of results. The preoperative preparation of anaesthetic drugs, anaesthesia implementation for upper GI endoscopy, and PACU (postanaesthesia care unit) clinical data collection and follow-up after the operation will be performed by three medical personnel to ensure that pharmaceutist, operators, recorders, and the patients are blind to group classification. On the day of study, an envelope with the smallest sequential number was opened first and the solutions of medicines were prepared in a syringe by an investigator based on the number appeared on the paper slip. Then, the unlabelled syringes of solutions were handed over to an anaesthesiologist who was performing general anaesthesia and administrating the medicines but was blind to the contents inside of the syringes. Another anaesthesiologist who observed and recorded the data intraoperatively and postoperatively was also blinded to the medication patient had received. All sedation procedures were standardised and performed by the same anaesthesiologist who was blind to the patient groups. PACU (postanaesthesia care unit) clinical data collection and follow-up after the operation were also blinded to the therapeutic regimen. The allocation sequence was not available to any member of the research team until databases had been completed and locked. An agreement from PI will allow unblinding of individual participants on a need-to-know basis and, if necessary, termination of treatment in the event the MSO and investigator determine the participant has had a serious adverse reaction to the study medication. Emergency unblinding can be performed at any time if it is considered necessary by the principal investigator. Participants are issued with “In case of emergency” cards to be carried at all times during the study including an emergency phone number. If the trial or a single subject is prematurely unblinded, the principal investigator will document the reason for unblinding and notify the ethics committee. Unblinding of the project statistician who will analyse the data will occur at the end of the study, after the last participant has been evaluated, all data have been entered and cleaned, and the database has been locked.

### Intervention

**Fig. 1:**
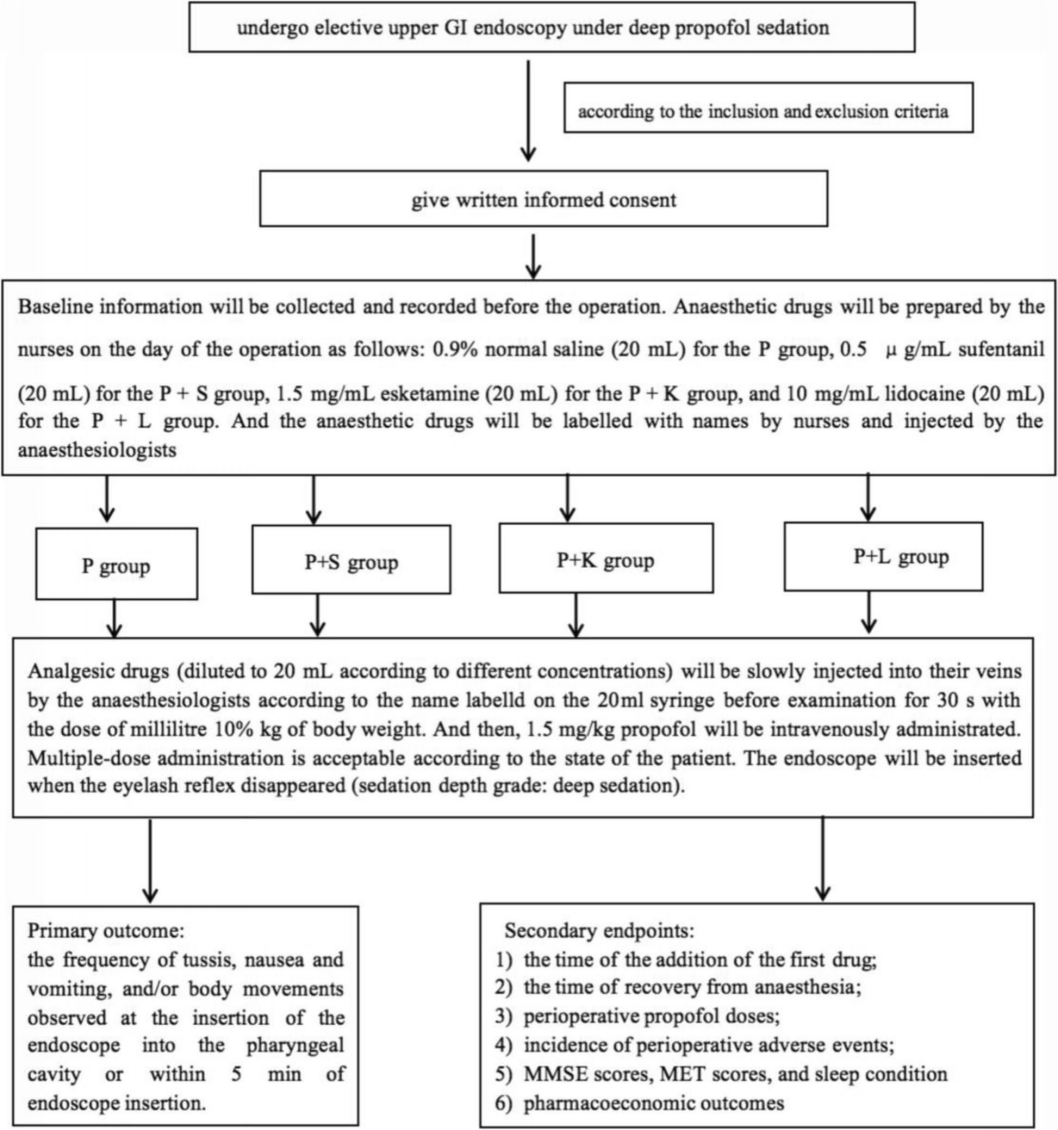
Study outline

Figure 1 presents the study outline. The patients are selected according to their inclusion and exclusion criteria for painless upper GI endoscopy and informed consent. The patients will be divided into the four groups: P group (single administration of propofol), P + S group (administration of propofol and sufentanil in combination), P + K group (administration of propofol and esketamine in combination), and P + L group (administration of propofol and lidocaine in combination) (*N* = 100 per group). Baseline information will be collected and recorded before the operation. Anaesthetic drugs will be prepared by the nurses and anaesthetists on the day of the operation as follows: 0.9% normal saline (20 mL) for the P group, 0.5 μg/mL sufentanil (20 mL) for the P + S group as a mixture, 1.5 mg/mL esketamine (20 mL) for the P + K group, and 10 mg/mL lidocaine (20 mL) for the P + L group. Before entering the operating room, the patients will be given lidocaine defoamer for gargling to open the upper limb vein and 5 mL/min lactate Ringer solution. After entering the room, the patients will wear masks to inhale high-flow oxygen, and their vital signs will be monitored and recorded as Tire. Analgesic drugs (diluted to 20 mL according to different concentrations) prepared by the nurses will be slowly injected into their veins 5 min before examination for 30 s with the dose of millilitre 10% kg of body weight, and then, 1.5 mg/kg propofol will be intravenously administrated. Multiple-dose administration is acceptable according to the state of the patient. The endoscope will be inserted when the eyelash reflex disappeared (sedation depth grade: deep sedation). Blood pressure will be recorded after every 3 min from the beginning of the examination, and HR, SpO_2_, and RR will be recorded simultaneously. The primary outcome was the frequency of tussis, nausea and vomiting, and/or body movements observed at the insertion of the endoscope into the pharyngeal cavity or within 5 min of endoscope insertion. When subclinical respiratory depression (90% ≤ SpO_2_ < 95%) occurred, the jaw-thrust manoeuvre was performed to open the airway. When hypoxia (75% ≤ SpO_2_ < 90% for less than 60 s) occurred, in addition to the jaw-thrust manoeuvre, the oxygen flow rate was increased from 2 to 6 L/min. In case of any abnormalities, notifications should be given, and appropriate doses of propofol should be added until the endoscope exits the teeth pad. During endoscopy, 2–4 mL of propofol should be added under the conditions of extended operation time, accelerated breathing, and elevated blood pressure and heart rate to maintain deep sedation. The patients will be transferred to a recovery room after the operation, and their vital signs will be recorded after every 5 min until the patients met the standard of leaving the hospital (Steward score ≥ 4) before discharge. For each patient, the anaesthesia protocol will be completely decided by an investigator based on their experience and expertise and clinical practice guidelines. The intervention was conducted by experienced health professionals under the supervision of researcher groups. In addition, randomly check was performed to make sure the adherence of the staff to the intervention protocols. There were no concomitant interventions that are prohibited during this trial. There is no compensation or post-trial care for participants.

### Assessments

The pain intensity, cognition function, insanity, anxiety, and depression in the patients will be assessed using a standardised and validated questionnaire. Before enrolment, the patients will be treated in anaesthesia evaluation clinics and screened according to the inclusion criteria and informed consent (Additional file 1). The demographic characteristics of the patients (age, gender, height, weight, medical history, medication history, allergy history, ASA grade, and sleep condition) will be collected, and physical characteristics, blood pressure, heart rate, and oxygen saturation of blood will be measured and recorded as Tbase. Preoperative examinations (urinalysis, haematological examination, and electrocardiogram) will be conducted to validate cardiac, liver, kidney, and coagulation functions. Pain intensity will be evaluated using the numerical rating scale. Anxiety and depression will be assessed using hospital anxiety and depression scale (HADS). Cognition function was assayed through mini-mental state evaluation (MMSE).

On the day of the operation, the vital signs, including blood pressure, heart rate, pulse oxygen saturation, and respiratory rate, will be measured and recorded when the patient entering the operating room and after induction and endoscope entrance, Afterwards, these signed will be recorded every 3 min until the patient left the room. The drug doses of anaesthesia, administration route, anaesthesia time, operation type, operation time, times of biopsy, infusion quantity, blood loss, and satisfaction of endoscopist (satisfied, average, or dissatisfied) will be documented. The vital signs of the patients in the recovery room will be recorded after every 5 min. MMSE scores will be re-evaluated before the patient leaves the recovery room, and the patients will be asked about their satisfaction degree (satisfied, average, or dissatisfied) and the operation and pain severity (0 point represents absolute no pain; 10 points denote unbearable pain). The incidence of adverse events (dizziness, headache, cardiopalmus, dyspnoea, mobility, nausea, and vomiting) will be followed up through phone calls after 0.5 h, 2 h, 1 day, 2 days, 3 days, 1 week, 1 month, and 6 months of the operation. The information about sleep conditions will be collected, and the alterations of MET scores, MMSE scores, HADS scores (the details of any specific manifestations will be inquired) will be assessed.

Adverse events will be assessed and treated throughout the study. When the perioperative oxygen saturation of blood < 95%, the anaesthesiologists will raise the lower jaw and open airway; this phenomenon is defined as insufficient oxygen supply. The condition of the oxygen saturation of blood of < 90% is defined as hypoxemia. When the oxygen saturation of blood < 88%, the endoscope will be removed, and the patient will be fitted with the mask for 100% pure oxygen inhalation and respiration will be assisted using a balloon. Simultaneously, adverse events, such as cough, laryngospasm, low and high blood pressure, bradycardia, and tachycardia triggered by drugs or operation, will be documented. Postoperative adverse events, including nausea, vomiting, dizziness, oversedation, infection, nightmare, diarrhoea, insanity, blurring of vision, nystagmus, and hallucination, will be recorded in detail.

### Endpoints

The primary endpoints include the frequency of tussis, nausea and vomiting, and/or body movements observed at the insertion of the endoscope into the pharyngeal cavity or within 5 min of endoscope insertion. Secondary endpoints are as follows: (1) the time of the addition of the first drug, from the end of induction to the addition of the first drug (from the injection of the study medication to the disappearance of eyelash reflex); (2) the time of recovery from anaesthesia, from the end of the operation till the patient left the recovery room (the end of operation to consciousness return); (3) perioperative propofol doses; (4) incidence of perioperative adverse events, including tachycardia, oxygen desaturation (the time of 90% ≤ SpO_2_ < 95% for more than 10 s), breathing inhibition (the time of SpO_2_ < 95% for more than 15 s), bradycardia (< 50 beats per minute or a decrease in HR of 20% or more from baseline), and high and low blood pressure (blood pressure < 30% of the basal blood pressure or systolic blood pressure or blood pressure > 30% of the basal blood pressure); (5) incidence of postoperative adverse events (within 6 months after the operation) including nausea, vomiting, increased secretion, dizziness, over sedation, infection, nightmare, uneasiness, itching, disorientation, insanity, breathing inhibition, diplopia, diarrhoea, intestinal obstruction, urinary retention, gastroesophageal reflux, constipation, shiver, and hallucination; (6) MMSE scores, MET scores, and sleep condition after 0.5 h, 2 h, 4 h, 1 day, 2 days, 3 days, 1 week, 1 month, and 6 months of the operation; (7) pharmacoeconomic outcomes (calculated incremental cost-effectiveness ratio based on cost-effectiveness analyses).

### Adverse events

In this study, a serious adverse event is defined as any of the following conditions: death, a life-threatening adverse event, inpatient hospitalisation or prolongation of existing hospitalisation, a persistent or significant incapacity or organ damages, a congenital anomaly/birth defect, and a significant medical event that requires intervention. The relationship between the adverse event and intervention will be determined and summarised by the investigators. Adverse events will be treated and reported to the institutional review board (IRB) as soon as possible. Study interventions will be treated for free. The trial will be terminated immediately in case of serious life-threatening events leading to prolonged hospital stay or death.

### Statistical methodology

Efficacy assessment is based on the intention-to-treat (ITT) population, which is defined as all randomised patients. Safety analyses will be performed in a safety evaluation set, which is a subset of all the patients exposed to a minimum of one dose of the studied medication. The SPSS 24.0 software will be used for statistical analyses. Significance will be analysed using two-sided tests, and *p* < 0.05 will be judged as the statistical significance standard. The normally distributed measurement data are presented as mean ± standard deviation (*x* ± *s*). The quartile method will be used to explain the measurement data with non-normal distribution. The repeated analysis of variance and Turkey test will be used for comparisons within a group and between multiple groups, respectively. Count data will be analysed using non-parametric tests, such as Rank-sum test, chi square test, and Fisher exact test. The multivariate regression analysis will be employed to explore the effects of different analgesic drugs combined with propofol on tussis during upper GI endoscopy. We will consider performing predefined subgroup analyses on the primary endpoints according to age, operation types, and special populations. Missing data will not be imputed. In addition, the primary endpoint analysis will also be conducted in a per-protocol set and compared with the ITT analysis to analyse sensitivity. No interim analyses are planned.

### Sample size calculation

Based on the results of previous pretest, we speculated that compared with only propofol, the combination of small doses of esketamine with propofol could exhibit significant effect against tussis reduction incidence by a minimum of 15% during upper GI endoscopy. The type I error probability, power of test, and sample size ratio will be set to 0.05, 0.8, and 1:1, respectively. According to relevant literature reports, some believe that a sample size of 18 patients per group will provide 80% of the ability to detect differences between groups 15% difference at a level of *α* = 0.05. At *α* = 0.05, based on the 80% power difference of 15% difference in propofol demand between the levels and detection groups, the power was increased 90%, and the value [200 (109) vs.128 (53)] was added to the sample size calculation formula (*n* = 2*[(*α* + *β*) *σ*/*δ*]^2). The sample size will be estimated by considering a sample loss rate of 10%. Then, the sample size was obtained.

### Data handling and record keeping

The personal details of each participant will be collected and stored digitally in a database. Data managers will manage and monitor the data to analyse abnormal values and missing data. The database will be locked after data collection. To ensure data completeness and accuracy, the locked database will be provided to the statistician, who is not a part of the study team and will conduct independent statistical analyses. The data will be stored in a locked place for 5 years.

### Ethical considerations, amendments and dissemination

This trial will be conducted in accordance with the Declaration of Helsinki. The informed consent and assent process is in line with the Good Clinical Practice guidelines. This study plan (protocol version 1.2) is approved by the ethics committee of Beijing Friendship Hospital, Capital Medical University, China (2022-P2-051–02). Any significant modifications, which may affect the study and potential benefits or safety of the patients, including the changes in study objectives, study design, patient population, sample sizes, study procedures, and significant administrative aspects, made in the study protocol or other study documents will be submitted to the local medical ethical committee for approval and require a formal amendment. All the study participants will be notified, and informed consent will be requested again when necessary. The amendment will be updated on the trial register website to ensure transparency. The results of this trial will be published in a scientific journal or presented at scientific conferences, regardless of the outcome.

## Trial status

The recruitment will start on October 1, 2022, and is expected to be completed on April 1, 2023.The trial will start recruiting the patients in the Beijing Friendship hospital, Capital Medical University. Recruitment at other centres will begin when ethical approval is available.

## Discussion

In recent years, for sedation, propofol combined with an analgesic has replaced its conventionally utilised combination with benzodiazepines and has become the standard for analgo-sedation for upper GI endoscopy with the advantages of improved sedation titration, short recovery time, and good patient tolerance and satisfaction. Esketamine offers the advantage of the minimisation of sedation side effects, making optimal use of the synergy concept while acting as an analgesic. Susanne Eberl et al. reported that the use of esketamine in patients during ERCP resulted in good sedation and analgesia quality [13]. However, some main questions remain unanswered. What are the optimal dose and drug compatibility recommended in clinical use? What is the efficacy of esketamine on tussis inhibition? What is the effectiveness of esketamine in the Chinese population? Can esketamine be well tolerated in actual clinical practice? Esketamine is the first esketamine biosimilar developed in China. Phase III studies in China have shown that esketamine with a shorter recovery and orientation recovery times than esketamine presents potential clinical advantages [16, 17]. Although its pharmacokinetics and safety have been reported preliminarily, the data were only from small-scale studies. Moreover, safety analyses in a clinical setting are limited. Therefore, we selected the RCT (randomised controlled trial) for numerous cases to explore the efficacy and safety of esketamine to obtain highly reliable evidence for the clinical application and risk management of esketamine.

Propofol is one of the most common drugs used in gastrointestinal endoscopy [18]. Compared with midazolam/pethidine, propofol has faster sedation induction and shorter half-life, and its use is increasing every year [19, 20]. Although propofol has its advantages, it has no analgesic effect. In addition, propofol sedation can easily provide a higher dose of general anaesthesia. Moreover, propofol is associated with circulatory and respiratory suppression, particularly in elderly patients [21, 22]. The combination of propofol and opioids is a commonly used anaesthesia method in bronchoscopy, although it is known that there are hypoxemia, postoperative nausea, and vomiting and other side effects, especially for patients without airway devices [23, 24]. Joskova et al. found in animal experiments that the pharmacological interaction between propofol, sufentanil, and midazolam mediated by GABAA receptors has a negative impact on the ciliary pulsation frequency of respiratory epithelial cells [25]. The affinity of s-enantiomer esketamine of ketamine racemic mixture to NMDA receptor is higher than that of r-enantiomer. Jiangsu Hengrui Medicine Co., Ltd., completed the preclinical study of Esquitan 3 years ago and obtained the clinical study approval from the State Food and Drug Administration [16]. Esketamine has sedative and analgesic functions. In addition, its sympathetic nervous system-like properties offset the hemodynamic inhibition of propofol, thereby reducing the risk of cardiovascular and respiratory depression. Hypotension is uncommon due to the increased tension of sympathetic nervous system and the maintenance of spontaneous breathing and airway reflex [26]. The combination of local pharyngeal anaesthetic (TPA) and intravenous sedatives may be helpful for endoscopic examination and improve the tolerance of patients by reducing vomiting reflex. Although the efficacy of adding TPA in intravenous sedation is still controversial, data from a clinical trials have examined the combination of lidocaine and propofol and showed that the use of lidocaine in conjunction with propofol during elective EGD might delay the time to discharge of patients. Given the fact that esketamine, sufentanil, or lidocaine combined with propofol showed better clinical outcomes, we recruited 400 patients and stratified them according to the operation type and age to acquire large-scale, randomised evidence for the safety and effectiveness to reduce the cough reflex of esketamine. Currently, American Society of Anesthesiologists (ASA) classification I–III will be randomised to four groups: P group (single administration of propofol), P + S group (administration of propofol and sufentanil in combination), P + K group (administration of propofol and esketamine in combination), and P + L group (administration of propofol and lidocaine in combination) (*N* = 100 per group). By this study design, the efficacy of these combinations will be evaluated.

In this trial, we plan to recruit 400 patients and stratified them according to the operation type and age to acquire large-scale, randomised evidence for the safety and effectiveness to reduce the cough reflex of esketamine.

Although the trial is designed carefully, some limitations remain persistent. First, we designed this RCT with the inherent limitation, the potentialcriticism for increased resources. Second, maybe the sample cannot exhibit good typicality due to theconcentrationofcasesinBeijing3Agradehospitals.Moreover, the questionnaires, which may be influenced by the patient’s perception of pain, are highly subjective; therefore, theymaynotdirectlyprovidetheeffect and lead to subjective bias. Thus, before beginning the trial, the investigator and site staff will receive systemic training for theuseofthequestionnairesand be certificated to prevent subjective bias to the highest possible extent.

In summary, this trial will be an important attempt to evaluate the efficacy and safety of esketamine in upper GI endoscopy. Moreover, in a large population and real settings, the proposed approach can be completely qualified for the selection of the optimal treatment for sedation in upper GI endoscopy.

### Supplementary Information

**Additional file 1. **Inclusion criteria and informed consent.

**Additional file 2. **SPIRIT checklist for trials.

## Data Availability

After the study is completed, the data will be open to the public through the Research Manager (ResMan) platform (http://www.medresman.org/login.aspx) within 6 months.
