# Supplementary material for: Comparison of the effects of esketamine, sufentanil, or lidocaine combined with propofol on tussis reflection during upper gastrointestinal endoscopy: study protocol for a randomised, two centre, three-blind, controlled trial
Source: Trials. 2024 Jan 4;25:24. doi: 10.1186/s13063-023-07812-0 (PMC10768256; doi:10.1186/s13063-023-07812-0)
Supplement: Supplementary file 1 — Additional file 1. Inclusion criteria and informed consent. [file 13063_2023_7812_MOESM1_ESM.doc]

**科研课题知情同意书**

方案名称：**比较艾司氯胺酮、舒芬太尼或利多卡因联合异丙酚对上消化道内镜诊疗时呛咳反射的影响:一项双中心随机三盲对照研究**

申办者**：首都医科大学附属北京友谊医院**

主要研究者：**侯海军**

尊敬的受试者：

您被邀请参加比较艾司氯胺酮、舒芬太尼或利多卡因联合异丙酚对上消化道内镜诊疗时呛咳反射影响的研究。请仔细阅读本知情同意书并慎重做出是否参加本项研究的决定。当您的研究医生或者研究人员和您讨论知情同意书的时候，您可以让他/她给您解释您看不明白的地方。我们鼓励您在做出参与此项研究的决定之前，和您的家人及朋友进行充分讨论。若您正在参加别的研究，请告知您的研究医生或者研究人员。本研究的目的、背景、研究过程及其他重要信息如下：

# 一．研究背景

内镜技术为上消化道系统疾病最常用的诊疗方法，在上消化道肿瘤的筛查及诊疗中有非常重要的临床价值。近年来随着麻醉技术的发展，以及患者对舒适化医疗服务需求的不断提高，麻醉下内镜诊疗的推广和普及也是必然的趋势，能够极大程度地减轻患者在诊疗过程中的不适。

众所周知，呛咳作为上消化道内镜诊疗中并不罕见的并发症，可增加气道痉挛，误吸，恶心呕吐等发生率。镇静剂和镇痛药对气道反射均有不同程度的抑制作用。目前异丙酚已被广泛推荐用于内镜检查的镇静，其起效快，作用时间短，对中枢神经系统有广泛的抑制作用，减少咳嗽、体动以及术后头痛的发生。但是异丙酚作为单一药物使用时，会出现镇静不足，抑制呛咳反应弱，临床疗效短等弊端，增加术后呼吸抑制及低氧血症的风险。既往研究提出异丙酚与阿片类药物联合使用可以增加镇静效果、抑制咽喉反射和应激反应，但静脉注射舒芬太尼引起的咳嗽并不少见，而且异丙酚与阿片类药物联合使用，对心血管系统和呼吸系统的抑制有协同作用，麻醉风险显著增加。既往亦有研究显示较低剂量的艾司氯胺酮即可减少呛咳发生率，且耐受性良好。当其与异丙酚联用时，既可以抵消异丙酚对循环和呼吸的抑制作用，又可以减少异丙酚的用量，在临床日渐普及，但是艾司氯胺酮具有拟交感作用，能一过性升高血压和心率，与内镜置入刺激咽喉时的交感反射叠加，进一步增加心脏负荷，不利于潜在心肌缺血的患者。静脉注射利多卡因能抑制气管机械性刺激黏膜上交感神经感受器的功能，预防呛咳反应的发生。但是其心肌抑制作用明显，且局麻药中毒的风险个体差异很大，安全性有待进一步明确。

目前在上消化道内镜诊疗中镇痛和镇静的最佳方法仍存在争议，因此找到一个合适的镇静/镇痛方案是很重要的，我们推测较单独应用异丙酚，异丙酚与低剂量舒芬太尼、艾司氯胺酮或者利多卡因联合应用于上消化道内镜诊疗可降低呛咳的发生率，特别是异丙酚与艾司氯胺酮联合时可能更为合适。 为了验证我们的假设，我们设计了本临床研究，旨在研究不同药物与异丙酚联合应用对抑制内镜检查中呛咳反射的影响。

**二．研究目的**

本研究的主要目的是比较单独应用异丙酚，小剂量舒芬太尼、艾司氯胺酮或者利多卡因联合异丙酚作用下呛咳的发生率和程度。呛咳的诊断标准为:上消化道内镜置入咽腔时或者置入后 5min 内患者出现呛咳、恶心呕吐和/或体动反应。

**三．研究过程**

**1. 多少人将参与这项研究？**

研究对象选取分别在首都医科大学附属北京友谊医院和首都医科大学附属北京天坛医院就诊，2022年3月至2022年12月期间拟行无痛上消化道内镜诊疗的患者

**2. 研究步骤**

- 医生将询问记录您的病史，并进行体格和实验室检查，如果您是合格的纳入者，并且自愿参加本研究，请您签署这份知情同意书。
- 确定您可以参加本研究后，采用随机数字表法将患者分为P组：异丙酚单药组；P+S组：异丙酚联合舒芬太尼组；P+K组：异丙酚联合艾司氯胺酮组；P+L组：异丙酚联合利多卡因组；根据患者体重以及术中维持麻醉深度的需要给予麻醉药物，并严密监测患者生命体征，予以及时对症处理。
- 本研究的内镜操作医师都是由经验丰富操作熟练，已聘用主治医师三年以上的内镜医师进行检查。检查结束后，您将接受术后1天，1周，1月，6月的随访，包括镇痛评分、精神状态评分，睡眠评分，满意度和舒适度等调研。

**3. 这项研究会持续多久？**

该研究从术前一天到出院后随访，一般持续半年。您可以在任何时间选择退出研究而不受到任何惩罚，也不会丧失您本应获得的任何利益。然而，如果在研究途中您决定退出本研究，我们鼓励您先和您的医生商议。考虑到您的安全性问题，有可能在退出后，会进行一次相关检查。

**四．风险与受益**

**1. 参加本研究的风险或不良反应是什么？**

参加本研究可能给您带来的风险如下。常见不良反应有低血压、恶心、呕吐、心动过缓、感觉异常、头痛、头晕、高血压、寒战、心动过速、少见的不良反应主要是过敏反应，焦虑、抑郁状态，口角发麻，耳鸣。

**2. 参加研究有什么受益？**

本次调研旨在探索旨在为上消化道内镜诊疗患者镇静镇痛方式提供参考资料，加快其术后恢复进程，减少患者不良事件的发生率。已有小样本研究证实，小剂量艾司氯胺酮联合异丙酚应用于无痛上消化道内镜诊疗中的可行性和安全性，因此，参与本试验的受试者在试验过程中可能直接获益，且研究结果可能有助于该类受试者未来获得更舒适、更安全的医疗服务。

**五．除了参加本研究(或如果不参加此研究)，我还有没有可选的其他医疗方案？**

除了参与本研究，您有如下选择方案：

非麻醉方案

请您和您的医生讨论一下这些及其他可能的选择。

在您和其他受试者的理解和协助下，通过本项目研究的结果可能会在医学杂志上发表。但是我们会按照法律的要求为您的研究记录保密。研究受试者的个人信息将受到严格保密。除非应相关法律要求，您个人信息不会被泄露。必要时，政府管理部门和医院伦理委员会及其它相关研究人员可以按规定查阅您的资料。

**六．关于研究费用及相关补偿**

**1. 研究所用的药物及相关检查费用**

本研究不会对您的治疗采取任何形式的干预，不会给您带来潜在的额外花费。 因此，治疗费和检查费是由您自己负担的。

**2. 参加研究的补偿**

在本研究过程中发生的任何实验相关损伤，您都会获得免费的药物治疗，但不会获得额外的经济补偿。如果您在参与该研究项目的过程中产生与研究项目不直接相关的生病或受伤，那么这种治疗产生的费用由您自己承担。 医生将尽全力预防和治疗由于本研究可能带来的伤害。

**3. 发生损伤后的补偿**

如果发生与该项研究相关的损伤，您可以获得由首都医科大学附属北京友谊医院以及首都医科大学附属北京天坛医院提供的必要的医疗护理，经相关单位按中国有关法律进行认定后依照相关法律法规进行补偿/赔偿。

**七. 受试者权利**

在参加研究的整个过程中，您都是自愿的。如果您决定不参加本研究，也不会影响您应该得到的其他治疗。如果您决定参加，会要求您在这份书面知情同意书上签字。您有权在试验的任何阶段随时退出试验而不会遭到歧视或受到不公平的待遇，您相应医疗待遇与权益不受影响。

如果您出现严重的不良反应，或者您的研究医生觉得继续参加研究不符合您的最佳利益，他/她会决定让您退出研究。无需征得您的同意，资助方或者监管机构也可能在研究期间任意时刻终止研究。如果发生该情况，我们将及时通知您，您的研究医生也会与您讨论您拥有的其他选择。

**八．受试者责任**

作为受试者，您需要提供有关自身病史和当前身体状况的真实情况；告诉研究医生自己在本次研究期间所发现的任何不适；不得服用医生已告知的受限制药物、

食物等；告诉研究医生自己最近是否参与其他研究，或目前正参与其他研究

**九. 如果我有问题或困难，该与谁联系？**

如果您有与本研究相关的任何问题，请在工作日联系侯海军医生，电话*（18612568228）*，在下班时间、周末或者节假日请通过*（18612568228）*联系侯海军医生。

如果您有与自身权利/权益相关的任何问题，或者您想反映参与本研究过程中遭遇的困难、不满和忧虑，或者想提供与本研究有关的意见和建议，请联系首都医科大学附属北京友谊医院生命伦理审查委员会，联系电话：010-63139017

# 研究者告知声明

“我已告知该受试者**比较艾司氯胺酮、舒芬太尼或利多卡因联合异丙酚对上消化道内镜诊疗时呛咳反射的影响**的研究背景、目的、步骤、风险及获益情况，给予他/她足够的时间阅读知情同意书、与他人讨论，并解答了其有关研究的问题；我已告知该受试者当遇到与研究相关的问题时可随时与侯海军医生联系，遇到与自身权利/权益相关问题时随时与友谊医院生命伦理委员会联系，并提供了准确的联系方式；我已告知该受试者他/她可以退出本研究，我已告知该受试者他/她将得到这份知情同意书的副本，上面包含我和他/她的签名。”

获得知情同意的研究人员签名 日期

# 受试者知情同意声明

“我已被告知**比较艾司氯胺酮、舒芬太尼或利多卡因联合异丙酚对上消化道内镜诊疗时呛咳反射的影响**的研究的背景、目的、步骤、风险及获益情况。我有足够的时间和机会进行提问，问题的答复我很满意。我也被告知，当我有问题、想反映困难、顾虑、对研究的建议，或想进一步获得信息，或为研究提供帮助时，应当与谁联系。我已经阅读这份知情同意书，并且同意参加本研究。我知道我可以在研究期间的任何时候无需任何理由退出本研究。我被告知我将得到这份知情同意书的副本，上面包含我和研究者的签名。”

受试者签名 日期

受试者联系电话

**（当受试者知情同意能力欠缺或不足时，增加或替换以下方式：）**

法定代理人签字 日期
